# Overview in Machine-Learning-Assisted Sensing Techniques for Monitoring COVID-19

**DOI:** 10.3390/mi17030283

**Published:** 2026-02-25

**Authors:** Yan Feng, Ming La

**Affiliations:** 1 School of Artificial Intelligence, Pingdingshan University, Pingdingshan 467000, China; 6103@pdsu.edu.cn; 2 School of Chemistry and Environmental Engineering, Pingdingshan University, Pingdingshan 467000, China

**Keywords:** COVID-19, biomarkers, sensing techniques, electrochemical biosensors, optical biosensors, machine learning

## Abstract

Viruses suddenly emerging from obscurity or anonymity affect our quality of life and increase incidence rate and mortality. A typical example is the global coronavirus disease 2019 (COVID-19) pandemic. Although severe acute respiratory syndrome coronavirus 2, known as the pathogen of COVID-19 has been significantly eliminated, its monitoring is still crucial, as the infectious disease may break out again. Therefore, it is necessary to develop simple and effective tools for monitoring COVID-19 and other diseases. Here, we summarize the progress of machine-learning-based biosensors in the monitoring and management of COVID-19. This article mainly includes three sections: machine learning algorithms, machine-learning-assisted biosensors, and challenges and future perspectives. We believe that this work is valuable for developing artificial-intelligence-based innovative analytical devices for healthcare monitoring and management of COVID-19 and other infectious diseases.

## 1. Introduction

Famous viruses suddenly emerged from obscurity or anonymity, raising concerns about their continued spread in populations from an immunological perspective [1]. Severe acute respiratory syndrome coronavirus 2 (SARS-CoV-2) was the causative agent of the global coronavirus disease 2019 (COVID-19) pandemic. Even though SARS-CoV-2 has been significantly eliminated, its monitoring still remains crucial, as the infectious disease may break out again [2,3]. Early assessment of viruses is crucial for clinical point-of-care. In medical diagnosis, the detection of viruses can be performed in the laboratory using traditional methods such as polymerase chain reaction (PCR) amplification and enzyme-linked immunosorbent assays [4], both of which require markers such as radioactive isotopes, enzymes, and fluorescent groups that can be easily measured by various analytical techniques. In addition, although these methods have extremely high sensitivity and selectivity, they typically require multiple detection procedures and skilled operators.

Compared with traditional analytical methods, biosensors have advantages such as fast response, low cost, non-destructive, and on-site detection [5]. They are widely used in basic biological research, food safety, environmental monitoring, disease diagnosis, and drug screening. In recent years, with the widespread development of nanotechnology, signal amplification strategies, and transducers, significant progress has been made in sensing fields [6,7]. However, most biosensors still rely on the use of antibodies or aptamers as biological receptors. In addition, they inevitably exhibit some irregular or high signal noise and have a short quality-guarantee period and poor stability, accuracy, and reliability, limiting their commercial value in practical applications [8]. Researchers are seeking new methodologies to improve the analytical performance of biosensors.

Artificial-intelligence-based data analysis brings great promise for developing new strategies to overcome the challenges of current biosensors, mainly including machine learning and deep learning [9,10]. Traditional biosensors can be developed into intelligent devices that can automatically predict the types and concentrations of analytes through decision-making systems [11]. The use of statistical or mathematical methods in chemometrics can explore more chemical information by analyzing the data and designing or optimizing the experimental and testing conditions [12]. Therefore, chemometrics can serve as an effective strategy or tool to address the issues of biosensors. The application of chemometrics in processing complicated signals obtained by various biosensors has been widely reported, and many advanced machine-learning algorithms are emerging. Compared with traditional methods, the advantage of advanced machine-learning methods lies in their ability to study appropriate nonlinear dependence in complex biological samples, providing unique possibilities for addressing urgent problems in the field of biosensors. This article aims to provide timely discussions and prospects of machine learning on the development of advanced biosensors for COVID-19 diagnosis.

## 2. Machine-Learning Algorithms for Biosensors

**Scheme 1:**
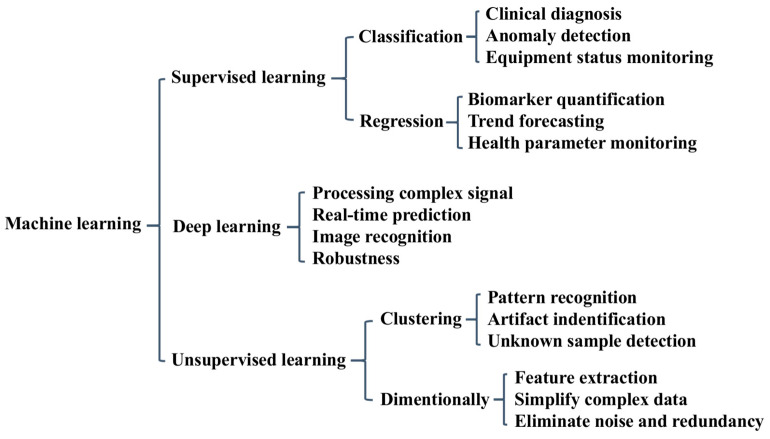
Machine learning algorithms and their functions.

Machine learning can effectively handle large-scale data from complex biological matrices. The major advantage of integrating machine learning with biosensors is that it can produce reasonable analysis results even in the case of high noise, low resolution, and potentially severe data overlap [13,14]. In addition, the correct application of machine-learning algorithms can reveal the potential relationships between sample parameters or biological events and sensing signals through data visualization. For the signal interpretation and performance optimization of biosensors, it is necessary to select appropriate machine-learning algorithms according to data characteristics such as linear/nonlinear, single component/multi-component, and signal-to-noise ratio. Scheme 1 shows an overview of machine learning algorithms in the application field of biosensing. Supervised, unsupervised, and deep-learning techniques each provide different functionalities, ranging from diagnosis and anomaly detection to pattern recognition and real-time prediction. As no algorithm is universally optimal, it is crucial to understand the principle and capability of various machine-learning methods in order to select the most suitable solutions and obtain the best results for specific sensing applications. This chapter briefly addresses several classical machine-learning algorithms used for disease diagnosis through the identification of biomarkers, mainly focusing on supervised and deep-learning methods.

Linear and logistic regressions are the two most fundamental linear models. They are suitable for simple detection systems where the signal is linearly correlated with the target concentration. The core function of linear regression is to construct a linear mapping relationship between feature parameter and target concentration, achieving quantitative analysis of a single component. The advantages of this model lie in its simplicity, high computational efficiency, and strong interpretability. Linear regression is suitable for the sample analysis with simple matrix and minimal interference, such as standard solution calibration, pure substance quantitative assay, etc. This method can replace traditional manual linear fitting curve with improved detection accuracy and efficiency. Logistic regression is a classification algorithm, which can achieve qualitative identification of target analytes based on different signal features. It is suitable for threshold detection scenarios and improves the accuracy of qualitative judgment by constructing a mapping relationship between the signal feature and the analyte format.

A support vector machine (SVM) is a versatile machine-learning model used for both classification and regression tasks. Its main advantage is to map linearly inseparable signal features to high-dimensional spaces through kernel functions, construct optimal classification hyperplanes, and achieve precise differentiation of complex signals. This model is particularly suitable for solving the problems of matrix interference and signal overlap in analytical chemistry. Some coexisting components in complex samples can easily generate interference signals, resulting in linear inseparability and making it difficult for traditional signal analysis methods to be distinguished. SVM can optimize the processing of radial basis kernel function, polynomial kernel function, etc., and explore the subtle difference in target/interference signals, thus achieving accurate separation of them and improving the selectivity of biosensors.

Random forest (RF) is an ensemble learning algorithm that can construct multiple decision-tree models and perform ensemble voting to achieve signal classification and regression. Due to its strong anti-interference ability and stability, the RF algorithm is suitable for long-term continuous monitoring scenarios in analytical chemistry. The core advantage of this algorithm lies in its insensitivity to outliers and its ability to effectively handle high-dimensional feature data. At the same time, it can evaluate the importance of each feature parameter to the detection results, providing a basis for optimizing the performance of biosensors. Random forest can effectively filter these abnormal signals and ensure the stability of detection results. In complex sample detection, the importance of feature parameters can be evaluated to screen out the signal features that are most sensitive to the target analyte, providing guidance for optimizing the recognition interface.

Artificial neural networks (ANNs), particularly convolutional neural networks (CNN) and recurrent neural networks (RNN) in deep learning, are effective algorithms for processing complex signals. They simulate the hierarchical structure of human brain neurons and achieve end-to-end analysis of raw signals without manually extracting feature parameters. When addressing challenges such as simultaneous detection of multiple components or low signal-to-noise ratios, deep learning can play a crucial role. By leveraging multi-layer network architectures to uncover deep features from raw data, the method enables precise separation and quantification of overlapping multi-component signals, showing robust noise-filtering capabilities. However, it requires a large amount of high-quality training data. Usually, this method is suitable for analysis scenarios involving multiple components, complex matrices, and high-precision detection.

## 3. Machine-Learning-Assisted Biosensors for COVID-19 Diagnosis

### 3.1. Electrochemical Biosensors

**Figure 1:**
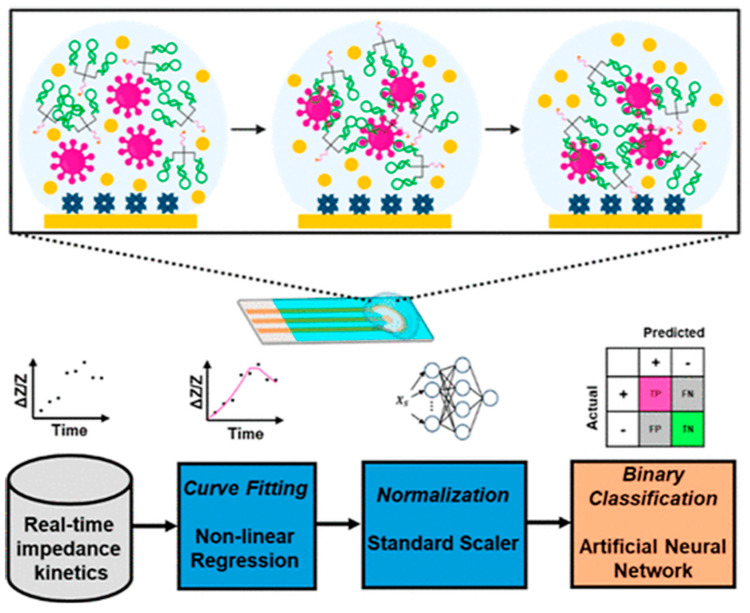
Schematic illustration of the real-time multimeric aptamer assay. Reprinted with permission from ref. [22]. Copyright 2025 American Chemical Society.

Although a comprehensive theory of electrochemistry can interpret various complex signals, electrochemical biosensors still exhibit poor reproducibility and stability in practical applications [15,16,17,18]. Real samples with many interferences may exhibit significant differences in ion strength, temperature, pH value, and others. The modified electrodes used in electrochemical biosensors typically become passivated over time. Therefore, one-dimensional data analysis alone is not sufficient to obtain sensitive signals highly correlated with the types and levels of analytes [19,20]. Machine-learning algorithms have been combined with electrochemical biosensors to improve the accuracy of COVID-19 diagnosis [21,22,23,24,25,26,27,28,29,30,31]. For example, Soleymani’s group developed an electrochemical method for the determination of SARS-CoV-2 virus through the interactions between the engineering trimeric aptamers (TMSA52) and the trimeric antigen spike proteins on the virus surface [21]. Many impedance data points for each sample have been obtained to deliver highly accurate test results in a short testing time with the assistance of machine learning algorithm. This method was successfully used to diagnose COVID-19 with a 100% precision by determining real human saliva samples from 27 negative donors and 10 positive donors. After that, Soleymani’s group designed a framework to facilitate the application of machine learning for diagnostic data collected from 172 COVID-19 saliva samples based on a real-time multimeric aptamer assay (Figure 1) [22]. In this work, nine key features from the transient signals were extracted through multiple nonlinear regression models. The features were applied to train three machine learning algorithms (SVM, ANN, and RF) based on a training/testing ratio of 75/25. Traditional receiver operating characteristic (ROC)-based classification achieved an accuracy of 83.6%, while machine learning-based models significantly improved the performance. The accuracy of SVM, ANN, and RF was 86%, 100%, and 100%, respectively. The values are higher than that achieved by the ROC curve. Among these machine-learning algorithms, the ANN model exhibited superior performance in handling complex and high-variance data and improved the diagnostic accuracy for point-of-care testing.

**Figure 2:**
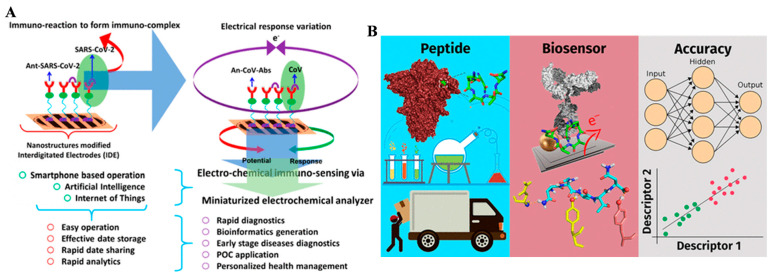
(**A**) Schematic illustration of electrochemical sensing of SARS-CoV-2 at point-of-care and artificial intelligence for intelligent COVID-19 management. Reprinted with permission from ref. [23]. Copyright 2020 American Chemical Society. (**B**) Schematic illustration of modular label-free electrochemical biosensor loading nature-inspired peptide toward the widespread use of COVID-19 antibody test. Reprinted with permission from ref. [24]. Copyright 2022 American Chemical Society.

Considering the challenges and aspects of COVID-19 management, Kaushik et al. explored the miniaturized point-of-care electrochemical biosensors for the determination of SARS-CoV-2 virus (Figure 2A) [23]. The diagnostics methods can be combined with artificial intelligence techniques such as machine learning and deep learning to investigate useful informatics through data storage, sharing, and analytics. In addition, Castro et al. reported a label-free biosensor by using a peptide Asn-Asn-Ala-Thr-Asn-COOH named PEP2003 as the recognition element to bind anti-SARS-CoV-2 spike protein antibody (Figure 2B) [24]. The peptide was noncovalently adsorbed on the gold nanoparticles (AuNPs)-coated carbon electrode and the signal change was recorded by electrochemical impedance spectroscopy. The binding of peptide and spike protein antibody was driven by hydrogen-bond and hydrophobic interactions. Through two equations fitted by machine learning, the biosensor was applied to diagnose COVID-19 with a 100% accuracy for 39 healthy and infected groups.

Electrochemiluminescence (ECL) is light production by an electrochemical reaction. It is a powerful tool for the determination of biomarkers with low background noise and high sensitivity. Firoozbakhtian et al. developed an ECL sensing system based on reverse transcriptase polymerase chain reaction (RT-PCR) cyclic threshold values [25]. The ECL signal change, dependent upon the concentration of SARS-CoV-2 virus, was monitored by a smartphone camera. With the ECL images to train a machine-learning algorithm, an ANN model obtained from the assays of 45 real samples exhibited > 90% accuracy for the diagnosis of 50 unknown samples and a cyclic threshold value of 32 for the determination of artificial samples.

**Figure 3:**
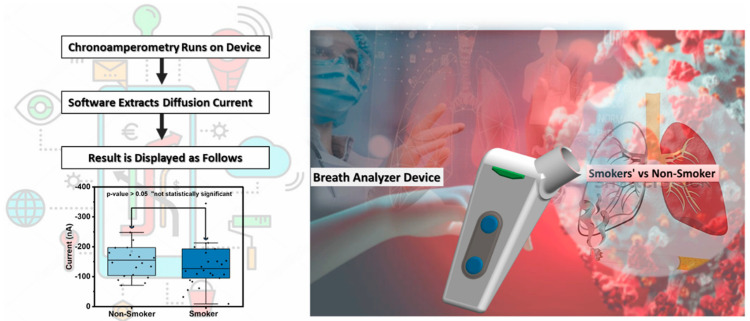
Working scheme for the breath analyzer platform. Reprinted with permission from ref. [26]. Copyright 2024 American Chemical Society.

Measuring the exhaled breath can offer a noninvasive method for monitoring the metabolic state of the human body. Banga et al. investigated the efficacy of a hand-held breathalyzer electrochemical sensing technology for predicting COVID-19 infection in the population of never or former light smoking history (Figure 3) [26]. The method was carried out based on the change in the exhaled nitric oxide level associated with COVID-19-linked respiratory inflammation. With this technology, a machine-learning algorithm was trained through the breath profiles of 46 infected and noninfected participants consisting of never or former light smokers. Both the technique and COVID-19 antigen rapid test were used for the assay of each participant. As a result, high specificity (91.11%) and negative predictive value (97.62%) were attained in the demographic groups. Although the technique can serve as a valuable tool for point-of-care COVID-19 diagnosis, large-scale clinical trials and validations are desired to prove its utility. The above works highlight new opportunities for combining machine learning with electrochemical biosensors to enhance their accuracy and reliability in actual sample analysis. However, different analytes require unique sensing materials, selecting and integrating compatible materials to achieve stable and accurate detection remains a complex and intensive task. Realizing long-term material stability can ensure consistent sensor performance over time, which is crucial for maintaining the reliability of diagnostic and monitoring systems in clinical and nursing environments. Thus, the practical application of machine-learning-based electrochemical biosensors is still in its infancy. Future research can focus on forecasting and improving material stability and quality guarantee period through machine learning, ensuring consistency and reliability of biosensor performance.

### 3.2. Optical Biosensors

#### 3.2.1. Colorimetric Methods

**Figure 4:**
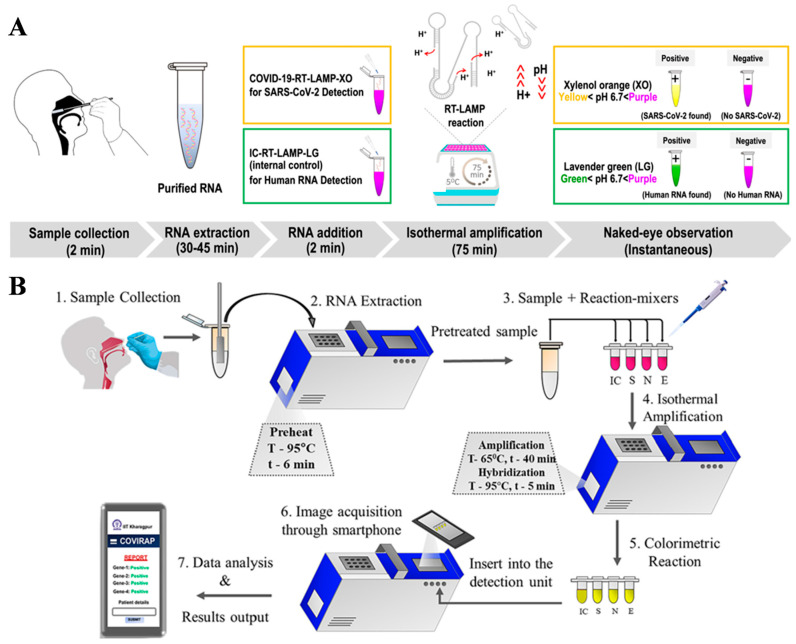
(**A**) Schematic illustration of the combined colorimetric COVID-19 assay with artificial intelligence-assisted automated result analysis. Reprinted with permission from ref. [40]. Copyright 2022 Elsevier B.V. (**B**) Schematic illustration of the sample-to-answer point-of-care detection process flow. Reprinted with permission from ref. [41]. Copyright 2023 Elsevier B.V.

Developing flexible optical biosensors has received widespread attention in view of their non-invasive nature, ease of observation, and good ability for continuous health monitoring. Such biosensors can significantly improve the accuracy and speed of disease monitoring, especially when integrated with artificial intelligence [32,33,34,35]. This combination opens up new opportunities in continuous health monitoring and personalized medicine. To date, highly sensitive detection of biological entities has been achieved through machine-learning-based optical biosensors such as colorimetry, fluorescence, and Raman scattering, and others (Table 1) [36,37,38,39]. Among them, colorimetric methods have shown great potential in COVID-19 diagnosis due to their high simplicity and low cost, especially in a low-resource setting [40,41,42,43,44,45,46,47]. However, most of the colorimetric methods for COVID-19 diagnosis involve specific pH-sensitive dyes, limiting downstream assay optimization or hindering efficient result interpretation. To resolve this problem, Kiatpathomchai’s group proposed a colorimetric real-time loop-mediated isothermal amplification (RT-LAMP) method for the assays of RNA by using dual dyes (Figure 4A) [40]. In this work, two in-house pH-dependent indicators (xylenol orange or XO and lavender green or LG) were used to improve sensitivity and simplicity. The colorimetric method was further combined with an artificial-intelligence-operated tool to achieve more precise and rapid assays in large-scale clinical trials. The method showed a detection limit of 50 viral copies/reaction with a cycle threshold value below 39.7, rendering it suitable for the point-of-care diagnosis of COVID-19. In addition, Biswas et al. reported a nucleic acid-based point-of-care RT-LAMP protocol for COVID-19 diagnosis with a machine-learning algorithm to improve efficacy [41]. As shown in Figure 4B, the detection procedures include seven steps: sample collection, RNA extraction, reactant addition, isothermal amplification, colorful reaction, imaging acquirement, and machine-learning-based data analysis.

**Figure 5:**
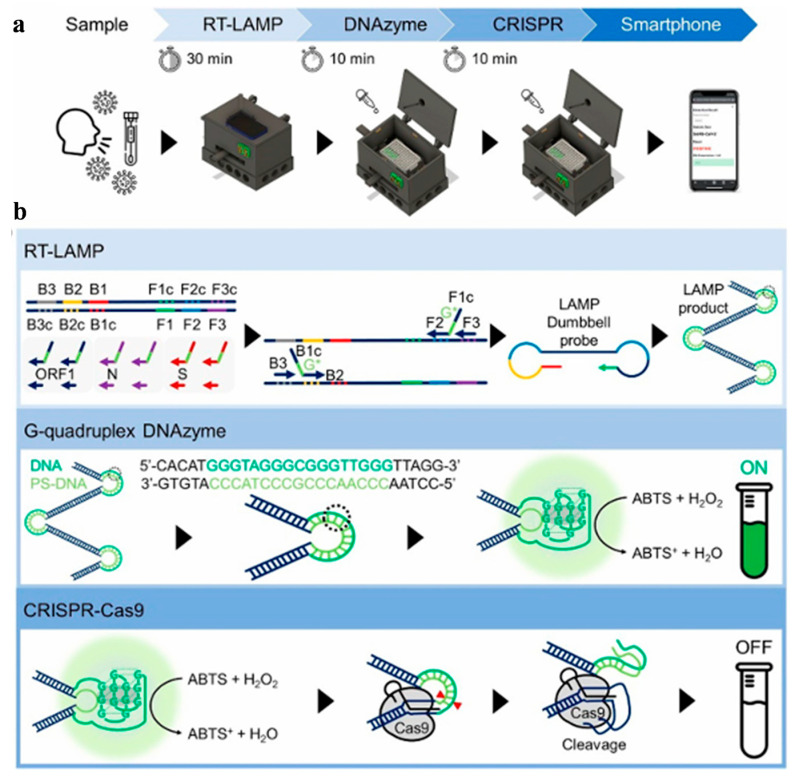
(**a**) Schematic illustration of DAMPR method for SARS-CoV-2 detection. (**b**) Principle of RT-LAMP, G-quadruplex DNAzyme, and CRISPR-Cas9 reactions. Reprinted with permission from ref. [42]. Copyright 2022 American Chemical Society.

The variant of SARS-CoV-2 can affect the accurate diagnosis of COVID-19. Song et al. reported a colorimetric LAMP-triggered DNAzyme reaction with the technique of clustered regularly interspaced short palindromic repeats (CRISPR) for monitoring SARS-CoV-2 and its variant genes (Figure 5) [42]. The CRISPR-associated system could eliminate the false-positive signal of LAMP product. This method showed attomolar sensitivity within one hour. In this work, a three-dimensional printing technique and a machine-learning-based smartphone application were used to collect the data and check the diagnostic results. For the test of 136 clinical samples, COVID-19 patients were diagnosed with 100% sensitivity and specificity. In addition, the method was successfully used to monitor three mutations of SARS-CoV-2 spike genes, including D614G (variant-common), T478K (delta-specific), and A67V (omicron-specific).

**Figure 6:**
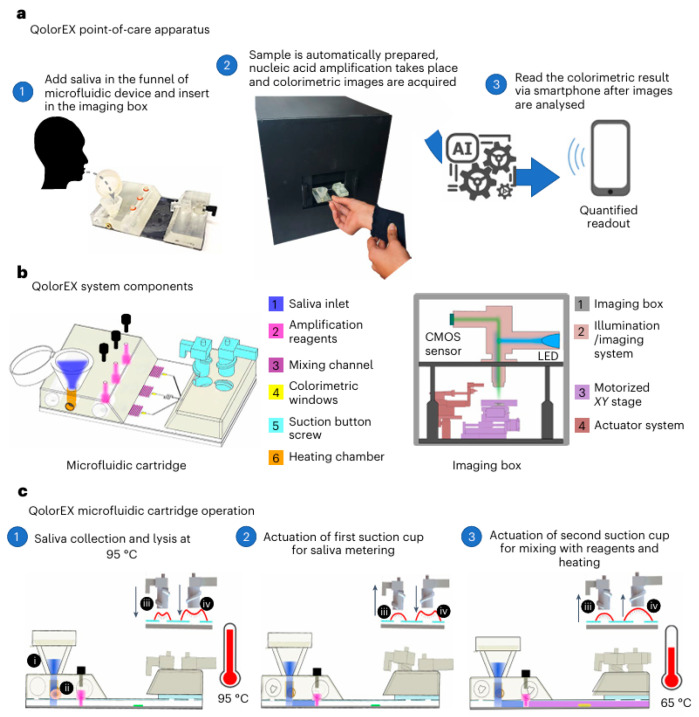
(**a**) Schematic illustration of QolorEX assay of respiratory infection-infected samples. (**b**) QolorEX system components. (**c**) microfluidic cartridge operation. Reprinted with permission from ref. [51]. Copyright 2024 Springer Nature.

In addition, Mahshid’s group presented a molecular diagnostic platform named QolorEX for determining SARS-CoV-2 and its variants by combining fabless plasmonic nano-surface into autonomous microfluidic cartridge (Figure 6). The device, composed of a microfluidic cartridge and an imaging box, was applied for the rapid and point-of-care identification of COVID-19 samples with machine-learning-assisted analysis. The microfluidic cartridge included six components: saliva inlet, amplification reagents, mixing channel, colorimetric windows, suction button screw, and heating chamber. The QolorEX microfluidic cartridge operation includes three steps: saliva collection and lysis at 95 °C, actuation of first suction cup for saliva metering, and actuation of second suction cup for mixing with reagents and heating. The method was successfully used to monitor COVID-19 saliva samples with a sample-to-answer time of 13 min and a 95% accuracy. Lateral flow immunoassays, paper-based vertical flow immunoassays, and other colorimetric test strips are the commonly used visual biosensors [48]. It is more attractive for clinical or home diagnosis to combine colorimetric biosensors with smartphone readers [49,50]. Smartphones and cloud-based machine-learning models may provide new avenues for high-precision and reproducible colorimetric analysis.

#### 3.2.2. Fluorescent Methods

**Figure 7:**
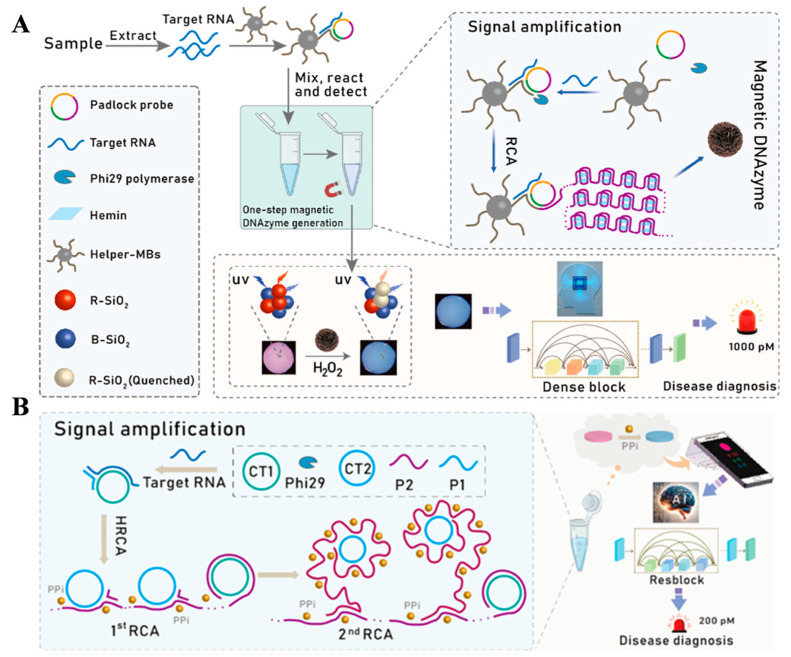
(**A**) Schematic illustration of workflow for the SARS-CoV-2 assay. Reprinted with permission from ref. [52]. Copyright 2024 Elsevier B.V. (**B**) Schematic illustration of machine learning-assisted platform for point-of-care testing of SARS-CoV-2. Reprinted with permission from ref. [53]. Copyright 2025 Elsevier B.V.

Fluorescent biosensors can achieve real-time, highly sensitive, and multiplexed detection due to their rapid response and strong signal intensity [52,53,54,55,56,57,58]. However, fluorescent biosensors currently used for early clinical diagnosis of COVID-19 are invasive, expensive, susceptible to interference from biological background fluorescence, and lack sufficient penetration capability and challenges related to imaging. The analytical performance of fluorescent biosensors can be improved by using machine-learning techniques to filter out noise from raw signals, extract relevant features, and fully decode complex parameters. The ensemble methods can classify signals into diagnostic categories such as positive, negative, or uncertain, and accurately distinguish the quantity of analytes. Wang et al. presented a machine-learning-assisted paper-based ratiometric fluorescence biosensor the determination of SARS-CoV-2 RdRp gene (Figure 7A) [52]. Target-induced rolling circle amplification was employed to produce magnetic DNAzymes, which could be monitored by the paper-based ratiometric fluorescence biosensor. The biosensor was fabricated by integrating blue-SiO_2_ (B–SiO_2_) and quantum dot (QD)-modified SiO_2_ nanoparticle/dopamine (DPA-QD@SiO_2_ or R–SiO_2_) into the cellulose paper. For the assays of target SARS-CoV-2 RdRp gene, the catalytic oxidation of dopamine into dopachrome by magnetic DNAzyme and H_2_O_2_ quenched the fluorescence of QDs on R–SiO_2_. Meanwhile, the corresponding fluorescent images were collected by the RNN machine learning platform. This method achieved the detection of SARS-CoV-2 RdRp gene with >99% accuracy and a detection limit of 30 fM. In addition, Wang et al. reported a RNN machine-learning-assisted ratiometric fluorescence biosensor for point-of-care testing of SARS-CoV-2 RdRp gene with metal–organic framework Al^3+^/Au NCs@ZIF-90 (Figure 7B) [53]. In this method, The ZIF-90 emitted blue fluorescence as a reference signal and the Al^3+^/Au NCs emitted red fluorescence as an analytical signal. The gene can induce hyperbranched rolling circle amplification (HRCA) to promote the production of pyrophosphate (PPi), leading to the quenching in the fluorescence of ratiometric paper biosensor. The detection limit was found to be 0.3 pM with an accuracy rate of over 99%. In addition, Samacoits et al. developed a smartphone-based device coupled with machine-learning-driven software to evaluate the fluorescence signal from the CRISPR diagnostic of COVID-19 [54]. The system showed a detection limit of 6.25 RNA copies/μL with 95% accuracy and 97% sensitivity for the assays of 96 nasopharyngeal swab samples.

**Figure 8:**
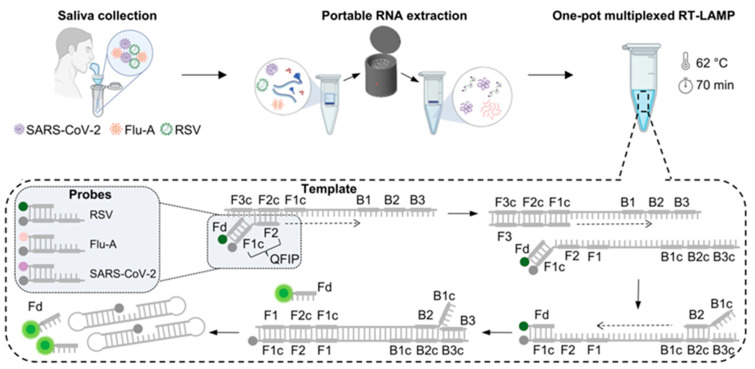
Schematic illustration of the test and the analyzer for multiplexed monitoring of RT-LAMP reaction. Reprinted with permission from ref. [55]. Copyright 2025 Elsevier B.V.

Accurate diagnosis of respiratory infections is particularly challenging when multiple pathogens have similar clinical symptoms. Kshirsagar et al. reported the simultaneous assays of three respiratory infections SARS-CoV-2, Influenza (Flu), and respiratory syncytial virus (RSV) by integrating RT-LAMP with a machine-learning-enabled compact analyzer [55]. As shown in Figure 8, the testing workflow includes three components: saliva collection, portable RNA extraction, and multiplexed RT-LAMP with specific primers and distinct fluorescent probes for one-pot multiplexed assays. The Forward Internal primer labeled with a quencher named QFIP was annealed to the fluorescently labeled probe named Fd complementary to the F1c region before the reaction was initiated. The fluorescence of the probe was quenched during the initiation of the F2 region. The hybridization of the F3 primer triggered the displacement of the quencher-labeled strand. As the backward internal primer (BIP) initiated the formation of the reverse strand, the Fd probe was separated from QFIP, resulting in the release of the fluorescence moiety. As more amplicons were generated in the LAMP reaction, the fluorescence signal increased until it reached a plateau. The method could determine three different RNA sequences with high accuracy. The area under the curve values for the assays of saliva samples were 0.82, 0.93, and 0.96 for RSV, Influenza, and SARS-CoV-2, respectively. The results are in good agreement with those achieved by RT-PCR assays.

**Figure 9:**
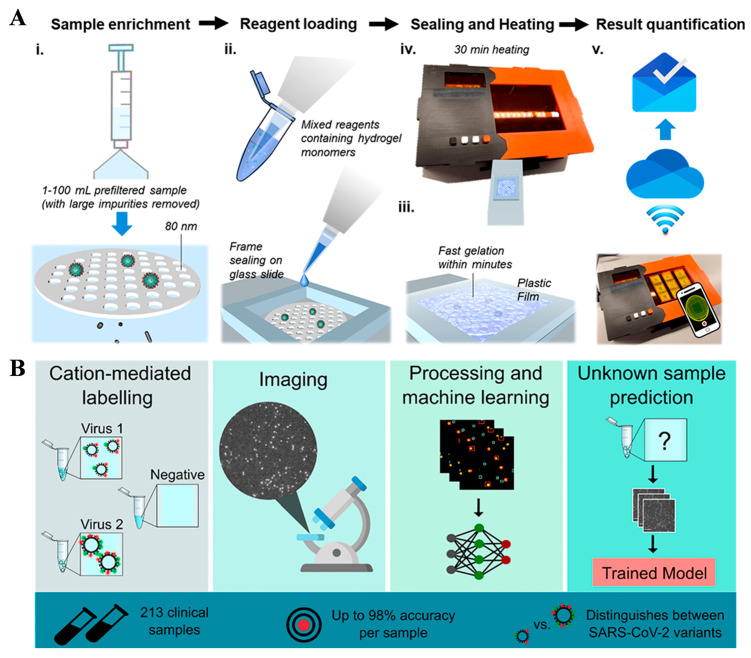
(**A**) Schematic illustration of mgLAMP workflow: (i) filtration, (ii) reagent loading, (iii) sealing, (iv) incubation, (v) imaging and result reading. Reprinted with permission from ref. [56]. Copyright 2021 American Chemical Society. (**B**) Schematic illustration of a fluorescently labeled and imaged strategy to detect viruses. Reprinted with permission from ref. [57]. Copyright 2022 American Chemical Society.

Monitoring viruses in ambient water is critical for environmental surveillance and early epidemic warning [59]. Digital polymerase chain reaction (dPCR) for fluorescence-imaging biosensors is a promising gene diagnostic technique. Accurately and quickly identifying positive reaction chambers in fluorescent images is crucial for the practical application of dPCR. Traditional methods such as threshold segmentation, numerical sequential clustering, and grid localization have been employed for image analysis. Zhu et al. developed a membrane-based LAMP system for the detection of SARS-CoV-2 virus in ambient water (Figure 9A) [56], including five steps: filtration, reagent loading, sealing, incubation, and imaging and result reading. This method could detect SARS-CoV-2 at a concentration down to 0.96 copies/mL in Milli-Q water and 93 copies/mL in surface water. The values are lower than those (930 copies/mL) obtained by RT-qPCR. The results were interpreted by smartphone and machine-learning-based imaging. The proposed method has significant value for large-scale environmental monitoring of SARS-CoV-2 without the use of professional equipment, trained personnel, and labor-intensive procedures. In addition, Shiaelis et al. reported a fluorescent method to detect and identify SARS-CoV-2 viruses using a convolutional neural network (Figure 9B). The viruses were fluorescently labeled, imaged and identified within 5 min. No lysis, purification, or amplification steps were required in this method. The proposed machine-learning algorithm could differentiate SARS-CoV-2 viruses from negative clinical samples and other common respiratory pathogens (e.g., influenza and seasonal human coronaviruses). This work suggested that the single-particle imaging technique could be combined with machine-learning algorithms to provide a promising alternative to the classical virus-diagnostic and gene-sequencing methodologies.

#### 3.2.3. Surface-Enhanced Raman Spectroscopy (SERS)

**Figure 10:**
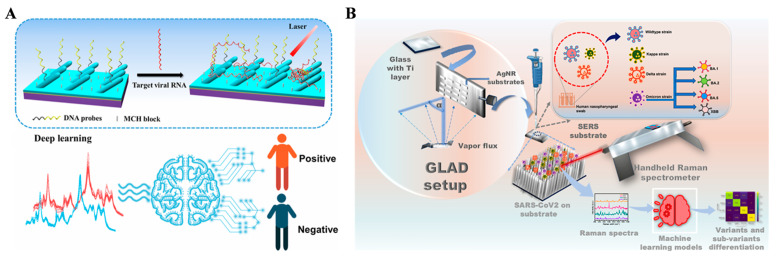
(**A**) Schematic illustration of direct NHS detection using a DNA SERS sensor and a deep-learning algorithm. Reprinted with permission from ref. [62]. Copyright 2022 American Chemical Society. (**B**) Schematic illustration of SARS-CoV-2 variant detection and differentiation using GLAD-pristine silver nanorod arrays and machine learning enhanced SERS. Reprinted with permission from ref. [63]. Copyright 2025 American Chemical Society.

SERS can obtain intrinsic fingerprint information of analytes in complex matrices. SERS biosensors are one of the most promising analytical tools for rapid, label-free, on-site, and non-destructive testing. However, many analytes and substances in the matrix have similar or overlapping spectra. This makes it almost impossible for SERS biosensors to directly distinguish targets. The combination of machine learning with SERS biosensors can greatly improve the effectiveness of target recognition [60]. The uniformity of enhancing factors for SERS substrates is crucial for machine-learning algorithms, since large variances in the dataset can increase prediction variance, making them only suitable for semi-quantitative or quantitative detection of COVID-1 [61,62,63,64,65,66,67,68,69,70,71,72,73]. Yang et al. reported a SERS biosensor for the determination of SARS-CoV-2 RNA in human nasopharyngeal swab (HNS) specimen through RNN-based deep learning (Figure 10A) [62]. A DNA probe specific to the RNA sequence was immobilized on the Ag-nanorod array (AgNR) surface. Binding of RNA to the DNA-modified AgNR surface led to changes in the SERS spectra. By using an RNN-based deep-learning method, 40 positive and 120 negative specimens were classified with a 98.9% accuracy. For the classification of 72 casual specimens, the method showed 97.2% and 100% accuracy prediction for the positive and negative specimens, respectively. Thus, the AgNR array-based SERS biosensor integrated with deep learning can be used as a potential platform for COVID-19 diagnosis. In addition, Senapati et al. developed a machine-learning-based SERS method for distinguishing SARS-CoV-2 in clinical HNS samples (Figure 10B) [63]. Glancing angle deposition (GLAD)-pristine AgNR substrate was prepared through a glancing angle deposition strategy and applied for the differentiation of the wildtype and variant of SARS-CoV-2. The detection limit was 100 pfu/mL for the assays of four variants and four covariants of the viruses. To distinguish the subtle spectral variations, machine learning was integrated with the SERS data to identify the complex patterns and enhance the diagnostic efficacy. In this work, two different classification methods, SVM and bidirectional long short-term memory network (BiLSTM) were used to identify the variants from 122 positive HNS samples. The accuracy was found to be 88.79% for SVM and 85.98% for BiLSTM. For the blind testing, the accuracy of SVM and BiLSTM was 74.77% and 70.09%, respectively. The integrated machine-learning-SERS biosensors could enhance diagnostic efficacy and provide on-site prediction ability for COVID-19.

**Figure 11:**
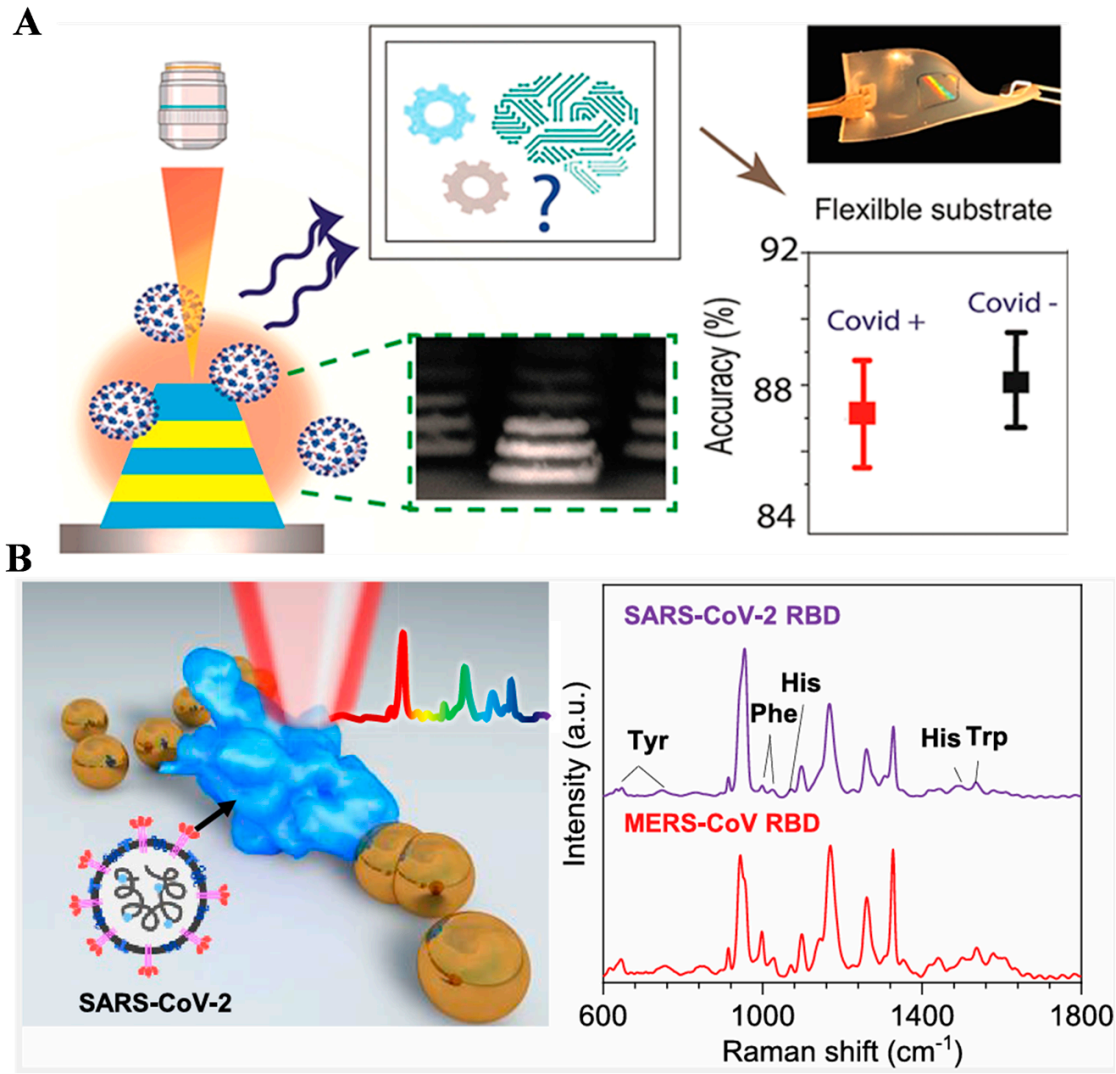
(**A**) Schematic illustration of SERS biosensor for distinguishing different types of respiratory and nonrespiratory viruses. Reprinted with permission from ref. [66]. Copyright 2022 American Chemical Society. (**B**) Schematic illustration of SARS-CoV-2 RBD SERS on AuNPs measured under a 785 nm laser. Reprinted with permission from ref. [67]. Copyright 2022 American Chemical Society.

Portable and label-free spectroscopy platforms will provide important tools for virus detection and sudden epidemic prevention. Paria et al. reported a machine-learning-assisted SERS biosensor for SARS-CoV-2 detection with metal–insulator–metal nanostructures (Figure 11A) [66]. The nanostructured substrate was prepared through nanoimprint lithography and transfer printing. The biosensor can distinguish different respiratory and nonrespiratory viruses within 25 min. It could also distinguish viruses in human saliva without any sample pretreatment. The nanopatterning approach indicated that biosensors could be developed on a flexible surface to develop wearable devices. Considering that the receptor-binding domain (RBD) of the SARS-CoV-2 spike protein is a key component of viral infection, Zhang et al. investigated the Raman property of SARS-CoV-2 RBD on the surface of AuNPs (Figure 11B) [67]. It was found that the Raman enhancement was strongly dependent upon the excitation wavelength due to the aggregation of AuNPs. The characteristic RBD Raman spectra for SARS-CoV-2 and MERS-CoV were determined by principal component analysis by revealing the secondary structure in the SERS spectra. Raman spectra of the two RBDs could be easily distinguished based on machine learning algorithms. The accuracy, precision, recall, and *F*_1_ scores were found to be all over 95%. This work is evaluable for rapid discrimination of complex proteins of infectious viruses and other biomolecules.

**Figure 12:**
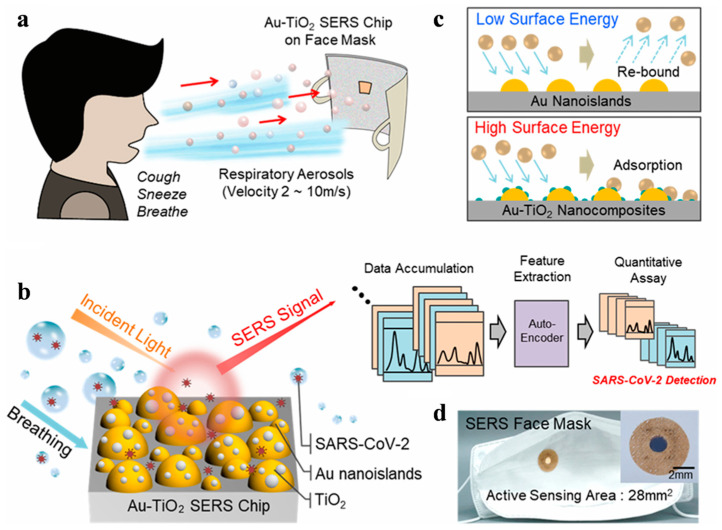
Schematic illustration of SARS-CoV-2 detection from respiratory breath aerosols using the Au-TiO_2_ SERS chip on a face mask: (**a**) oronasal aerosols, (**b**) machine-learned SERS detection, (**c**) aerodynamic behavior of aerosols, (**d**) optical image of the SERS face mask. Reprinted with permission from ref. [73]. Copyright 2022 American Chemical Society.

In addition. Hwang et al. reported a SERS biosensor for the detection of SARS-CoV-2 in respiratory aerosols (Figure 12) [73]. The biosensor consisted of a Au-TiO_2_ SERS face mask and an ablation-assisted autoencoder. The nanocomposite SERS face mask could continuously preconcentrate and efficiently capture oronasal aerosols. This substantially enhanced the SERS signal intensity by 47% compared to the simple Au nanoislands. The Au-TiO_2_ nanocomposite allowed for the successful determination of SARS-CoV-2 spike protein in artificial respiratory aerosols with a concentration down to 100 pM. The SARS-CoV-2 lysate at the concentration of 10^1^–10^4^ pfu/mL could be determined by using a deep-learning-based autoencoder to monitor the SERS feature of the spike protein. The detection range is comparable to that achieved by 19–29 PCR cyclic threshold assays for the samples of COVID-19 patients. The high accuracy (>98%) of this method indicated that the Au-TiO_2_ SERS face mask could provide a platform for determining various biomarkers in respiratory aerosols.

**Table 1:** Overview in machine-learning-assisted optical biosensors for COVID-19 diagnosis.

Biosensor	Biomarker	Performance	Algorithm	Dataset/Validation	Ref.
Color	RNA	LOD: 50 copies/reaction accuracy: 100%	DETR	213 human RNA samples 8:1 training/validation	[40]
Color	RNA	sensitivity enhanced by 21.15% accuracy: 99.26%	RF	406 human RNA samples 4:1 training/validation	[41]
Color	S gene	LOD: 1.08 aM accuracy: 99.38%	RF	216 samples 10-fold cross-validation	[42]
Color	RNA	sensitivity enhanced by 100-fold accuracy: 91.67%	deep learning	213 human RNA samples	[43]
Color	IgG and IgM	LOD: 55 pM (IgG) and 1.1 nM (IgG) accuracy: 100%	LDA	purchased serum 10-fold cross-validation	[44]
Color	SARS-CoV-2	LOD: 0.28 PFU/mL accuracy: 100%	RF	spiked saliva samples	[45]
Color	RNA	LOD: 4000 copies/mL accuracy: 95%	SVM	38 individuals 5-fold cross-validation	[51]
FL	RdRp gene	LOD: 30 fM accuracy: 99%	ResNet	21 RNA virus samples	[52]
FL	RNA	LOD: 6.25 copies/μL accuracy: 95%	Not reported	115 nasal swab samples 4-fold cross-validation	[54]
FL	RNA	LOD: 267 copies/reaction accuracy: 80%	neural network	14 spiked saliva samples	[55]
FL	SARS-CoV-2	LOD: 93 copies/mL high accuracy	AutoML Vision	wastewater samples 8:1:1 training/test/validation	[56]
FL	SARS-CoV-2	LOD: 6 × 10^4^ PFU/mL accuracy: 91.4%	CNN	7:3 training/validation	[57]
SERS	SARS-CoV-2	LOD: 10^5^ copies/mL accuracy: 100%	KNN and SVM	40 swab samples 5-fold cross-validation	[61]
SERS	RNA	LOD: 10^3^ copies/mL accuracy: 98.9%	RNN	160 specimens 7:3 training/test	[62]
SERS	SARS-CoV-2	LOD: 100 pfu/mL accuracy: 88.79%	SVM	122 nasal swab samples 4:1 training/validation	[63]
SERS	S protein	LOD: not reported accuracy: 100%	LR	65 swab and 70 negative samples 7:3 training/validation	[64]
SERS	RNA	LOD: 63 copies/mL accuracy: 90%	SVM	10 clinical samples	[65]
SERS	RNA	LOD: 500 nM accuracy: 83%	PCA, RF	pooled human saliva	[66]
SERS	RBD	LOD: not reported accuracy: 95%	PCA	unpurified or patient samples 5-fold cross-validation	[67]
SERS	SARS-CoV-2	LOD: not reported accuracy: 90%	RF	114 swab and 175 saliva samples 5-fold cross-validation 4/1 training/test	[69]
SERS	SARS-CoV-2	LOD: not reported accuracy: 85.5%	GPR	20 patient samples 10-fold cross-validation	[70]
SERS	S protein	LOD: 100 pM accuracy: 98%	SMOTE	500 respiratory aerosol samples 19:1 training/test	[73]

Abbreviations: FL, fluorescence; S protein, SARS-CoV-2 spike protein; RBD, receptor-binding domain of SARS-CoV-2 spike protein; LOD: limit of detection; DETR, detection transformer; RF, random forest; LDA, linear discriminant analysis; SVM, support vector machines; ResNet, residual networks; CNN, convolutional neural network; KNN, k-nearest neighbors; RNN, recurrent neural network; LR, logistic regression; PCA, principal component analysis; GPR, Gaussian process regression; SMOTE, synthetic minority oversampling technique.

## 4. Challenges and Future Perspectives

In summary, there is an urgent need to develop effective engineering tools for handling large datasets collected by biosensors and transferring laboratory diagnostics to personalized medical devices. In this work, we reviewed machine-learning-based sensing techniques for the prediction and diagnosis of COVID-19. Multiple biomarkers have been determined, and the datasets have been analyzed by various machine-learning algorithms. The qualitative identification of complicated overlapping signals and quantitative determination of low-abundance biomarkers have been upgraded. In addition, traditional data regression analysis uses mathematical equations to calculate the dependent variable of a sample, typically with fewer than two input features. In addition, advanced machine-learning models can handle databases with hundreds of input features. It is noticed that a sufficient dataset is crucial for machine-learning methods. The development of multi-channel or high-throughput sensing devices (e.g., microarrays and multi-channel fluid chips) can facilitate researchers to overcome the data bottleneck of integrating machine learning with biosensors. However, in the early stages of research, machine-learning-assisted diagnosis methods require a large amount of samples and data. Key issues such as data privacy, ethical considerations, and algorithmic biases require the implementation of robust encryption protocols, transparent data governance frameworks, and interpretable artificial-intelligence models. Future research should focus on overcoming these challenges by advancing adaptive learning systems, optimizing real-time data processing, and ensuring ethical and responsible use of powered biosensors.

Generalizability is another challenge in the clinical application of machine-learning models for COVID-19 diagnosis. In the reported models, the training datasets are well-representative samples of the clinical populations. However, in real-life screening scenarios, it is expected that the vast majority of patients or donors will be non-COVID-19 patients, resulting in a very imbalanced distribution of categories in the datasets. This highlights the necessity of implementing strict model-validation strategies to provide reliability measurements for each prediction based on individual characteristics and similarity in the model training, such as k-fold cross-validation, leave-one-out validation, and testing on an external dataset.

Compared with traditional methods used in laboratories, the reliability and accuracy of point-of-care testing are usually lower. The application of machine-learning algorithms in point-of-care testing provides an opportunity to improve the reliability and accuracy of biosensors for clinical analysis. Smartphones integrated with machine-learning algorithms may become be a very interesting home-testing tool in the further. For example, a mini-program can be created in a smartphone for data analysis to increase usability and convenience, in which an algorithm can be used to filter out abnormal frequency differences at the same concentration, fit the data into the model, and display the results on the screen. The analysis data for single-molecule, single-particle, and single-cell is challenged mainly by low signal-to-noise ratio, signal overlap, and signal dispersion. Traditional hypotheses based on data exploration and selection may not be reasonable, as unexpected signals may be missed. Using machine-learning methods to reduce noise and collect multiple signals can ameliorate the resolution of pattern recognition and the sensitivity of target detection.

Wearable biosensors have attracted significant attention due to their enormous potential for non-invasive human physiological monitoring through various biological fluids such as sweat, tears, and saliva. Combining wearable biosensors with machine-learning algorithms for health monitoring is another opportunity. The decisions of machines must be understood by professional staff. At the same time, it is necessary to incorporate human knowledge and reasoning rules into artificial-intelligence systems in a clear way, in order to reinforce and standardize the learning and decision-making processes and reduce the sample sizes for training algorithms. Therefore, it is urgently desired to combine interpretable machine learning with wearable electronic devices for health monitoring and related medical interventions.

## Data Availability

No new data were created or analyzed in this study.
